# The Vicious Cycle of Hypothyroidism and Severe Proteinuria: A Case Report

**DOI:** 10.7759/cureus.28674

**Published:** 2022-09-01

**Authors:** Shuojohn Li, Mahmoud Alsaiqali, Meenakshi Narayanaswamy, Isabel McFarlane

**Affiliations:** 1 Internal Medicine, State University of New York Downstate Health Sciences University, Brooklyn, USA; 2 Internal Medicine, State University of New York Downstate Medical Center, Brooklyn, USA

**Keywords:** thyroid, thyroid supplement, vicious cycle, proteinuria, nephrotic syndrome, hypothyroidism

## Abstract

Severe proteinuria and nephrotic syndrome are well documented causes or exacerbating factor of hypothyroidism. Less commonly known is that hypothyroidism, one of the most commonly encountered endocrinopathies, also has a profound effect on renal function and may lead to proteinuria. Here we report a case of female patient with hypothyroidism who presented with new onset severe proteinuria. Based on clinical data, we suspect the patient may have entered a vicious cycle of hypothyroidism and severe proteinuria where the two conditions worsen each other. Through literature review, we provide summaries of evidence supporting this bidirectional relationship between hypothyroidism and proteinuria, and emphasize on the importance of recognizing such association in clinical practice.

## Introduction

Hypothyroidism is one the most commonly encountered endocrinopathies clinically, affecting approximately 4.6% of the U.S. population [1]. While most cases can be readily managed with thyroid replacement therapy and carry a good prognosis, severe decompensated hypothyroidism (e.g., myxedema coma) remains a devastating disease and carries a mortality rate as high as 25%-60% even with treatment [2]. It is thus important to identify any potential precipitating factors that can destabilize thyroid function in patients with hypothyroidism. One such precipitating factor is severe proteinuria or nephrotic syndrome, which despite well-established association with thyroid dysfunction, is often missed by clinicians when approaching new onset or decompensated hypothyroidism. Conversely, there is a growing body of evidence supporting proteinuria as a presenting feature of hypothyroidism. Therefore, it is conceivable that if a patient presents with both hypothyroidism and severe proteinuria, such patient may enter a vicious cycle where the two conditions potentiate each other, leading to clinical decompensation. To avoid this potentially devastating decompensation, clinicians should recognize this bidirectional relationship between hypothyroidism and severe proteinuria as well as provide early intervention.

## Case presentation

A 53-year-old woman presented with progressively worsening abdominal distention and pain for three weeks prior to presentation. Patient also reported progressive shortness of breath of similar duration that was not associated with exertion. Review of systems revealed cold intolerance, nausea, dry eyes, dry mouth, and generalized itching. She denied all other constitutional symptoms otherwise, including vomiting, fever, and constipation. Comorbidities included current smoking, hypothyroidism, hypertension, diabetes mellitus, asthma, untreated hepatitis C, prior hepatitis B exposure and psychosis with delusion. On presentation, patient exhibited psychotic behavior and was not able to provide consistent information. Her home medication as per pharmacy report included levothyroxine, glipizide, alogliptin, and pravastatin. However, we were unable to confirm either the exact home regimen that she was on or adherence to the regimen.

**Figure 1:**
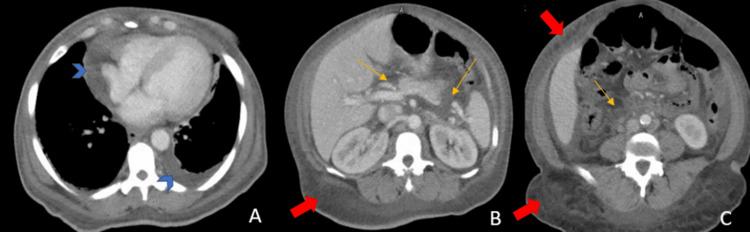
Abdominal CT result demonstrating extensive fluid overload Blue arrow head: pericardial effusion and pleural effusion, yellow arrow: abdominal ascites and periportal edema, red arrow: subcutaneous edema.

**Figure 2:**
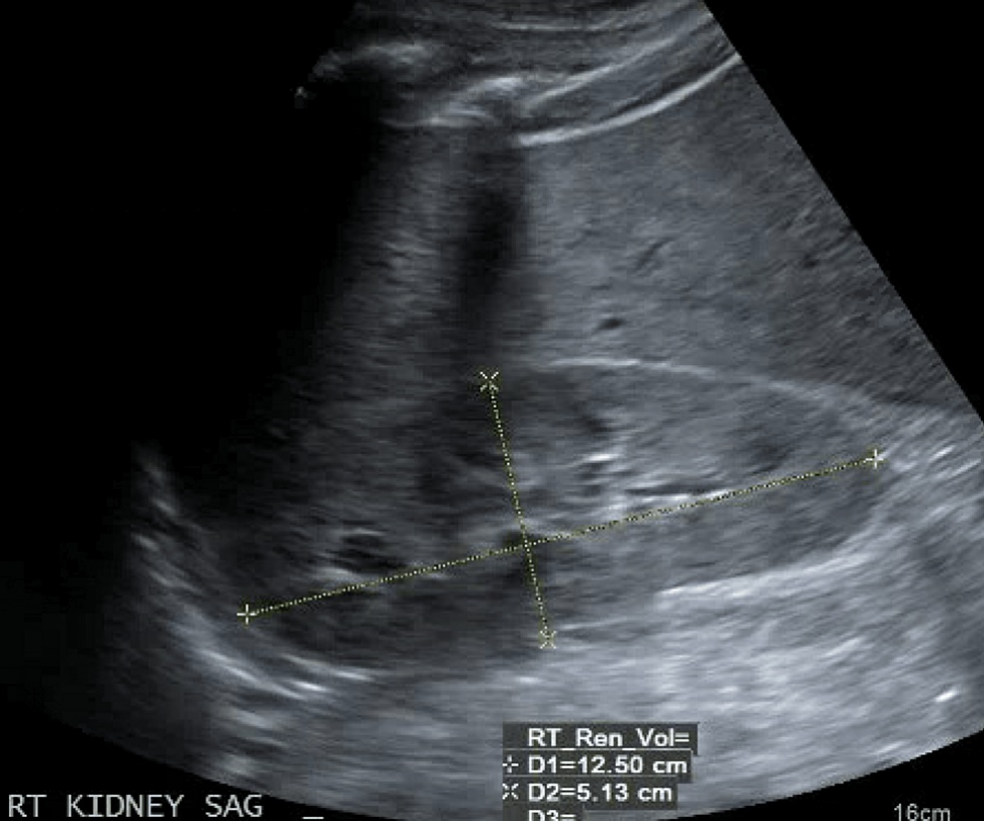
Renal ultrasound demonstrating increased echogenicity, suggestive of medical renal disease

On presentation she was afebrile, heart rate of 82, blood pressure of 163/97, respiratory rate of 22 with oxygen saturation of 94% on 2 liters of nasal canula. Physical exam revealed hoarse voice, periorbital edema and macroglossia. Bibasilar rales were heard on lung auscultation. Abdominal examination demonstrated positive fluid wave and shifting dullness. Bilateral 2+ non-pitting edema up to mid-calves were noted. The skin was dry, scaly associated with diffuse scratch marks. Computed tomography (CT) imaging showed diffuse fluid overload and anasarca, with pericardial effusion, pleural effusion, periportal edema, and ascites (Figure 1). Kidney ultrasound demonstrated increased echogenicity of the right kidney but no hydronephrosis, suggestive of medical renal disease (Figure 2).

Laboratory revealed white blood cells (WBC) of 7080/uL, hemoglobin of 9.5 g/dL, hematocrit of 31.4%, mean corpuscular volume (MCV) of 78.3 fL, platelet of 305,000/uL, thyroid stimulating hormone (TSH) of 47.25 uIU/mL, free thyroxine (T4) of < 0.4 ng/dL, creatine kinase (CK) of 258 units/L, albumin of 2.5 g/dL, creatinine of 1.1 mg/dL, blood urea nitrogen (BUN) of 13 mg/dL, cholesterol of 220 mg/dL, low-density lipoprotein (LDL) of 161 mg/dL, urine random protein of 104 mg/dL, urine random creatinine of 32 mg/dL, urine protein/creatinine ratio (UPCR) of 3.25. Urinalysis showed 30 red blood cells (RBCs) per high power field, dysmorphic RBCs without red blood cell casts (Table 1). Patient was given increasing doses of levothyroxine, and was diuresed with furosemide. Despite interventions, patient only experienced minimum symptom improvement, and her renal function deteriorated during the hospital stay (creatine was 1.8 mg/dL on hospital day four).

**Table 1:** Laboratory findings of our patient WBC: white blood cell, MCV: mean corpuscular volume, TSH: thyroid stimulating hormone, T4: free thyroxine, CK: creatine kinase, BUN: blood urea nitrogen, LDL: low-density lipoprotein

Laboratory test	Value	Reference range
WBC (10^3/uL)	7.08	3.5-10.8
Hemoglobin (g/dL)	9.5	12.0-16.0
Hematocrit (%)	31.4	37.0-47.0
MCV (fL)	78.3	78.0-98.0
Platelet (10^3/uL)	305	130-400
TSH (uIU/mL)	47.25	0.38-4.70
T4 (ng/dL)	<0.4	0.71-1.85
CK (units/L)	258	30-223
Albumin (g/dL)	2.5	3.5-5.7
Creatinine (mg/dL)	1.1	0.6-1.2
BUN (mg/dL)	13	7-25
Cholesterol (mg/dL)	220	0-200
LDL (mg/dL)	161	<99
Urine random protein (mg/dL)	104	Not established
Urine random creatinine (mg/dL)	32	Not established
Urine protein/creatinine ratio	3.25	<0.2

Further rheumatologic workup for possible glomerulopathy are summarized below (Table 2). At this point, the differential diagnosis remained broad including rapidly progressive glomerulonephritis (RPGN), membranous proliferative glomerulonephritis (MPGN), other glomerulopathies due to Sjogren’s syndrome, lupus nephritis, diabetic nephropathy, amyloidosis were also considered. A renal biopsy was recommended, however, the patient refused the proposed biopsy and signed out against medical advice on the fifth day of hospitalization. 

**Table 2:** Rheumatologic laboratory findings of our patient ANA: antinuclear antibodies, dsDNA: double-stranded DNA, GBM: glomerular basement membrane, RNP: ribonucleoprotein

Laboratory test	Value	Reference range
ANA titer	1:320	< 1:40
Complement C3	56	87-200
Complement C4	10	19-52
Thyroglobulin antibody (IU/mL)	>1000	<1
Thyroid peroxidase antibody (IU/mL)	756	<9
Anti-Smith antibody	Not detected	Not detected
dsDNA antibody (IU/mL)	<12.3	<30
Proteinase 3 antibody	Not detected	Not detected
Myeloperoxidase antibody	Not detected	Not detected
Anti GBM antibody	Not detected	Not detected
Anti RNP antibody	1.9	<1.0
C3 nephritic factor	Not detected	Not detected

## Discussion

Our patient’s presentation was concerning for glomerulopathy with mixed nephritic-nephrotic features. Her rheumatologic workups and dysmorphic RBCs on urinalysis raised suspicion of glomerulonephritis. However, she met all criteria for nephrotic syndrome, including severe proteinuria of > 3-3.5 by UPCR, hypoalbuminemia of < 2.5 g/dL, evidence of peripheral edema, and hyperlipidemia [3]. Despite the lack of pathologic diagnosis, this case highlights the importance of recognizing the bidirectional relationship between severe proteinuria and hypothyroidism.

Severe proteinuria or nephrotic syndrome have been shown to worsen thyroid functions. Peters et al. reported a significant inverse relationship between serum free triiodothyronine (fT3), serum free thyroxine (fT4) and the degree of proteinuria, as measured by UPCR, among the whole study cohort [4]. Interestingly, while the correlation between fT4 and proteinuria remained significant among patients with impaired renal function (defined by estimated glomerular filtration ratio (eGFR) < 60 ml/min), the correlation between fT3 and UPCR disappeared among this cohort [4], suggesting proteinuria may not be the only mechanism of how thyroid functions are affected by renal disease. Similarly, Yang et al. reported a significant negative correlation between serum fT4 and the degree of proteinuria, as measured by 24-hour urinary protein excretion [5]. Kwong et al. also reported that both the baseline TSH level and the odds of developing hypothyroidism were higher among patients with severe proteinuria of > 1.75g/day [6]. In addition, Karethimmaiah and Sarathi suspected that newly diagnosed nephrotic syndrome could destabilize patients with hypothyroidism who were previously on stable thyroid replacement regimen, increasing their serum TSH level and their thyroid replacement dosage requirement [7]. One proposed mechanism for the association between severe proteinuria and hypothyroidism is via the loss of thyroxine (T4) and triiodothyronine (T3) in urine [4]. This theory is consistent with the investigation by Chandurkar et al., who demonstrated the degree of proteinuria was positively correlated with the amount of urinary total T4 excretion [8]. Albeit this well-documented association, proteinuria and nephrotic syndrome are often not considered during the evaluation of new onset hypothyroidism.

Conversely, severe hypothyroidism can mimic symptoms and laboratory values that are seen in nephrotic syndrome, including reduced eGFR, proteinuria, dyslipidemia, and generalized edema. In a large cross-sectional study, Change et al. reported that both subclinical and overt hypothyroidism are associated with greater odds of developing decreased eGFR and proteinuria [9]. Weerakkody and Lokuliyana reported two cases where patients with severe hypothyroidism presented with proteinuria that gradually improved with thyroid replacement therapy [10]. In addition, Gondil et al. supported proteinuria as a presenting feature in severe hypothyroidism and found that in primary hypothyroidism patients without preexisting chronic kidney disease (CKD), thyroid supplement therapy resulted in lowering of creatinine and proteinuria as well as increased eGFR [11]. Similarly, Blackaller et al., reported in a randomized control trial that among patients with proteinuric CKD who are already on angiotensin-converting enzyme inhibitors (ACEIs) or angiotensin receptor blockers (ARBs), levothyroxine supplementation may decrease proteinuria and improve eGFR [12]. These findings suggest hypothyroidism may be a reversible cause of proteinuria and renal dysfunction. Finally, as reported by Khalid et al., myxedema ascites is a rare but well-recognized complication of hypothyroidism that may mimic the generalized edema seen in nephrotic syndrome [13]. Although the exact pathogenesis of myxedema ascites is still not fully understood, Parving et al. reported that low levels of thyroid hormone may lead to increased capillary permeability and protein extravasation, as well as decreased lymphatic drainage [14], all of which may contribute to the formation of myxedema ascites.

Because of this bidirectional relationship between hypothyroidism and severe proteinuria, we postulate that our patient had entered a vicious cycle where the two conditions were potentiating each other. Her presentation began with new onset renal dysfunction and severe proteinuria, resulting in generalized edema and thyroid function destabilization. As her thyroid function deteriorated, her symptoms and laboratory values were further exacerbated by the profound hypothyroidism. However, our interpretation of the case is limited by the lack of further laboratory and pathologic workup, which could potentially provide guidance to our inquiry. Despite limitations, we are able to highlight the intricate association between hypothyroidism and severe proteinuria through this case. Given that there is evidence supporting proteinuria as a presenting feature [9-11], clinicians should consider hypothyroidism as a cause when investigating sudden new onset proteinuria, along with other common etiologies (e.g., diabetes, HIV, amyloidosis, hepatitis, etc.). In addition, thyroid replacement therapy should be promptly started if hypothyroidism is identified since it may improve proteinuria and renal function [10-12]. Importantly, thyroid function needs to be rechecked regularly to titrate the dosage of therapy. Conversely, in patients with hypothyroidism that are previously on stable replacement therapy, an increase in TSH or thyroid replacement demand may warrant urine studies to check for proteinuria [7].

## Conclusions

In conclusion, here we report a patient with pre-existing uncontrolled hypothyroidism presenting with generalized anasarca and new onset severe proteinuria. Because of her clearly documented laboratory abnormalities and clinical presentations, we were able to seize this opportunity to explore the complex interrelationship between hypothyroidism and severe proteinuria. However, our interpretation of the case is limited by the lack of follow-ups and pathologic results. Future studies should focus on how treatment for hypothyroidism improves proteinuria and renal dysfunction, and vice versa. In addition, the association between hypothyroidism and proteinuria needs to be recognized in clinical practice. In patients with new onset proteinuria, thyroid function should be checked and hormonal supplementation started if elevated TSH is encountered. On the other hand, clinicians should interpret thyroid function tests with care in patients with severe proteinuria.
